# Supplementary material for: Clinical Testing for Mismatch Repair in Neoplasms Using Multiple Laboratory Methods
Source: Cancers (Basel). 2022 Sep 20;14(19):4550. doi: 10.3390/cancers14194550 (PMC9559284; doi:10.3390/cancers14194550)
Supplement: Supplementary file 1 [file cancers-14-04550-s001.zip › cancers-1836268-supplementary/SupplementaryDataYanget2022/Figures S1-S4.pdf]

Figure S1

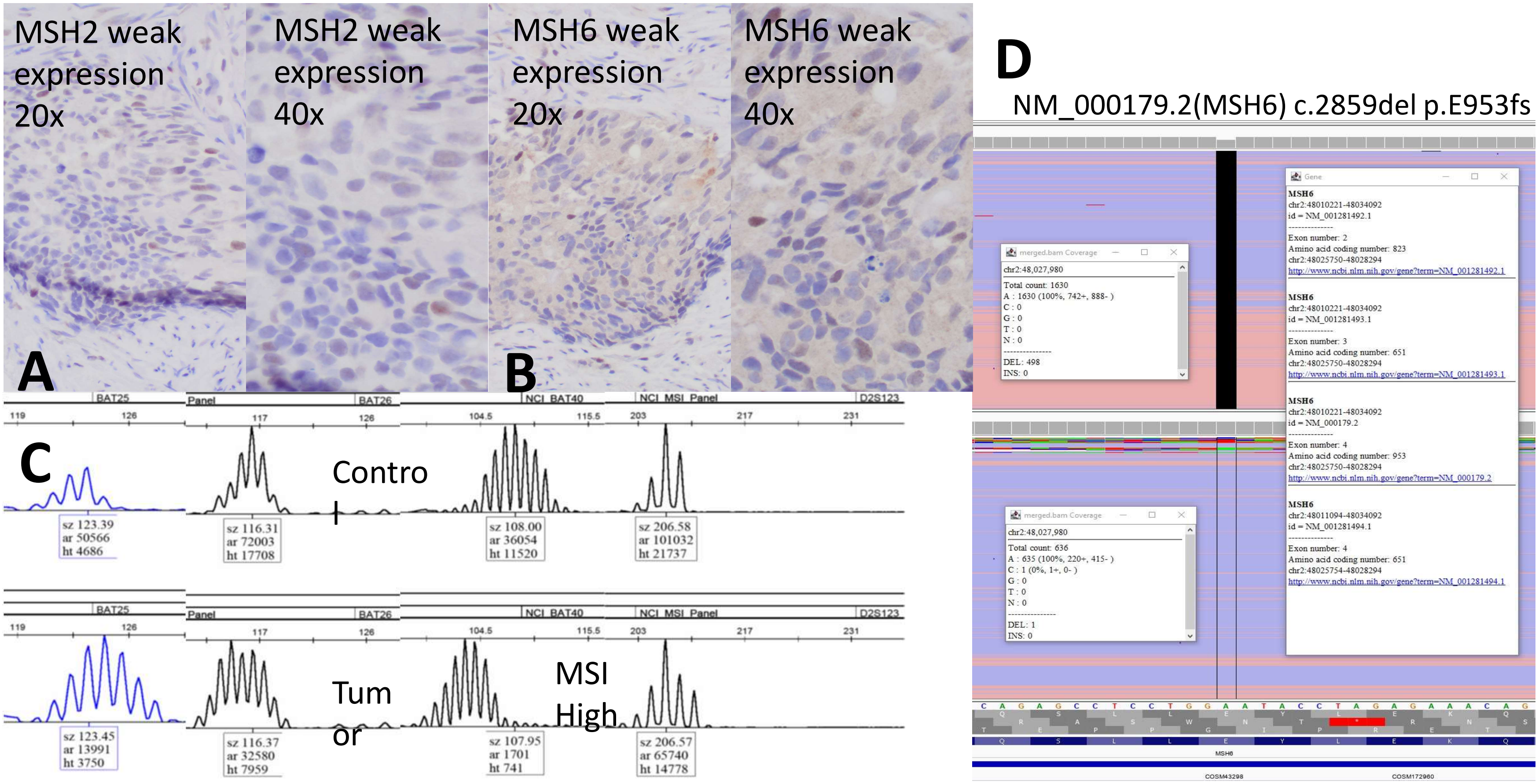

Figure S1. Case #36, Prostatic Adenocarcinoma, Minor Discrepancy, Two MSH6 somatic mutations, One germline MSH6 mutation, MSI-H, IHC MSH2, MSH6 weak expression. A) MSH2 4x Weak protein IHC staining. B) MSH6 20x Weak protein IHC staining. C) MSI-High, traces showing 4 shifted loci (BAT25, BAT26, BAT40, D2S123). D)IGV Trace showing somatic variant NM_000179.2(MSH6) c.2859del p.E953fs

Figure S2

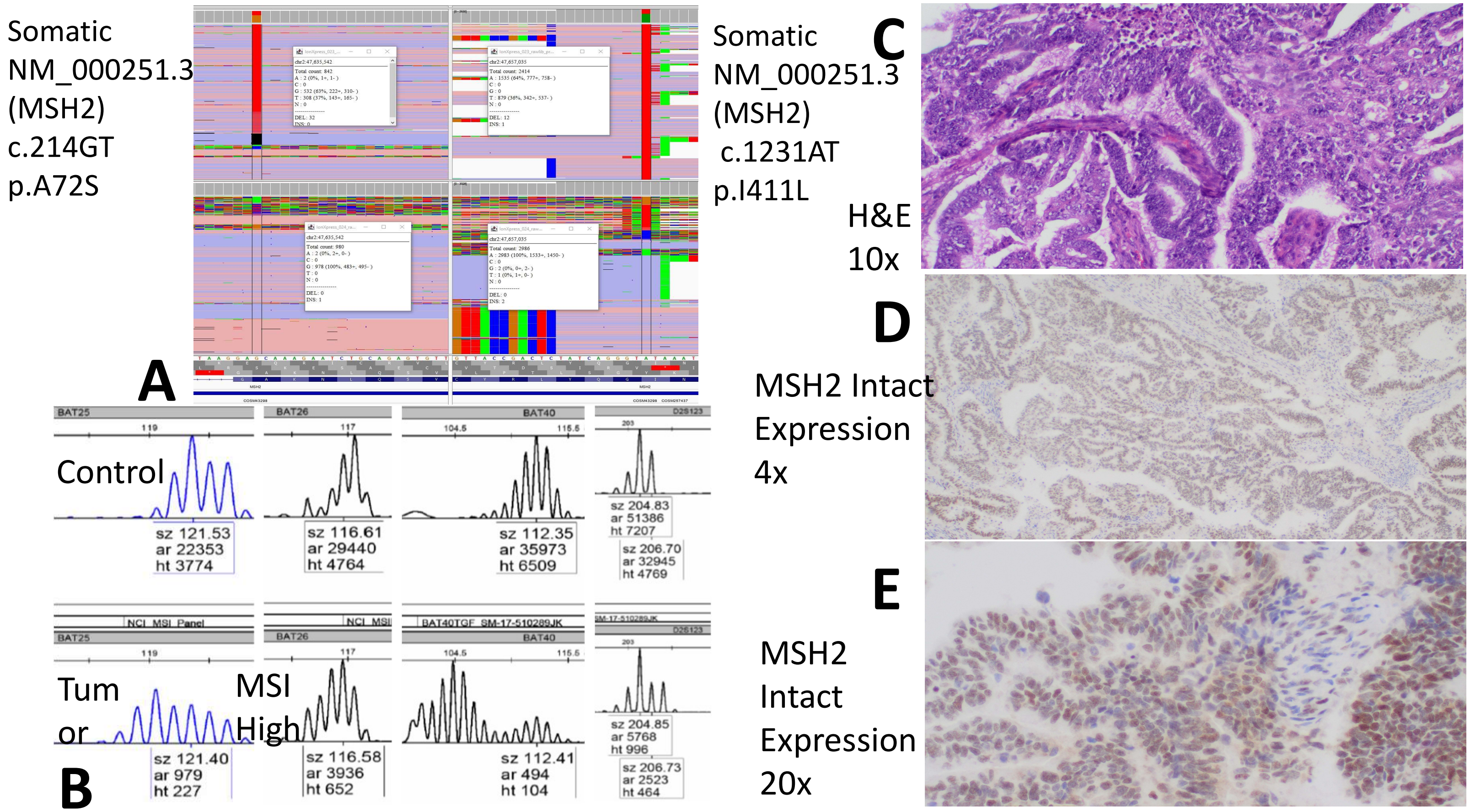

Figure S2. Case #265, Endometrial Carcinoma, Minor Discrepancy, Two MSH2 somatic mutations, MSI-H, but no IHC loss. A) IGV Trace showing somatic variants NM_000251.3(MSH2) c.214GT p.A72S and NM_000251.3(MSH2) c.1231AT p.I411L. B) MSI traces showing 4 shifted loci (BAT25, BAT26, BAT40, D2S123). C)H&E 10x Endometrial Endometrioid Adenocarcinoma MSH2 4x Intact MSH2 protein IHC staining. D)MSH2 20x Intact MSH2 protein IHC staining

Figure S3

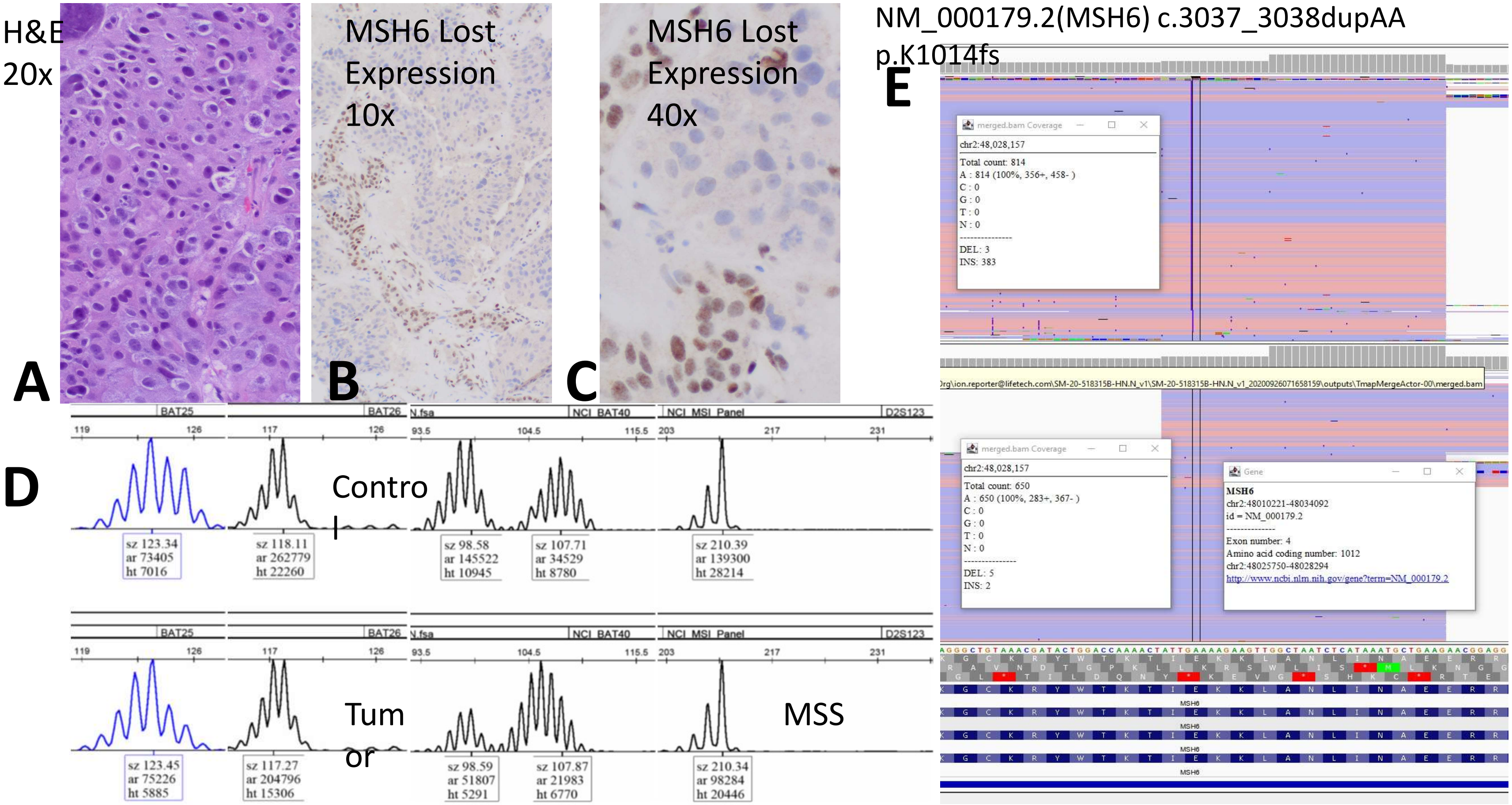

Figure S3. Case #271, Major Discrepancy, High Grade Neuroendocrine Carcinoma, Liver, S-20-072318 A1, NM_000179.2(MSH6) c.3037_3038dupAA p.K1014fs, Somatic Null Pathogenic mutations, Isolated Loss of MSH6 Staining, MSS Stable. A) H&E High Grade Neuroendocrine Carcinoma, Liver, 20x. B) MSH6 Isolated Loss of Protein IHC staining 10x. C) MSH6 Isolated Loss of Protein IHC staining 40x. D) MSS (Microsatellite Stable), traces showing 4 non-shifted loci (BAT25, BAT26, BAT40, D2S123). E) IGV Trace showing somatic variant NM_000179.2(MSH6) c.3037_3038dupAA p.K1014fs

Figure S4

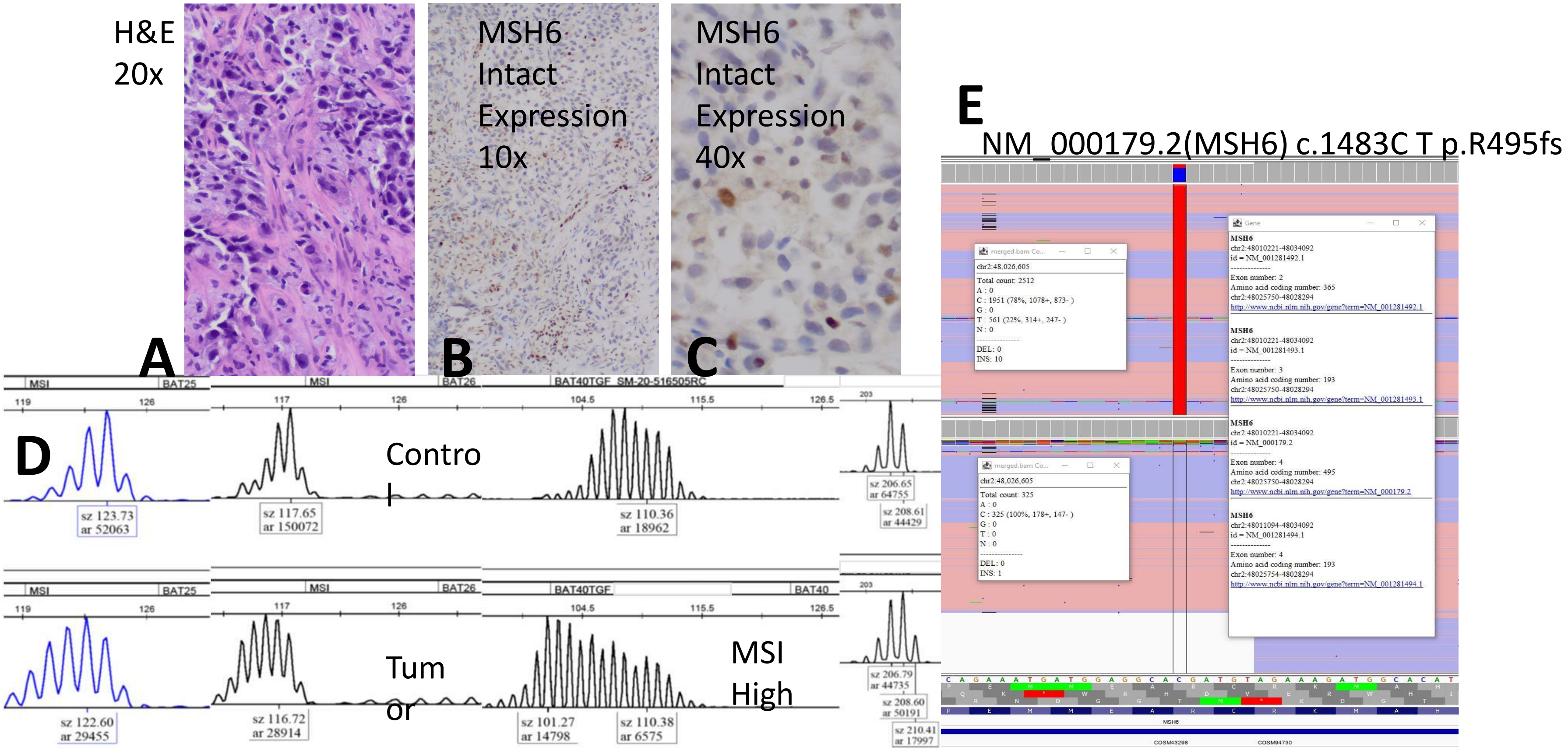

Figure S4. Case #659, Urothelial Carcinoma NM_000179.2(MSH6) c.1483C T p.R495 Major Discrepancy, One MSH6 somatic Nonsense mutation, One germline MSH6 frameshift mutation, MSI-H, IHC Expression Intact. A) H&E Urothelial Carcinoma 20x. B) MSH6 Intact Protein IHC staining 10x. C) MSH6 Intact Protein IHC staining 40x. D) MSI-High, traces showing 4 shifted loci (BAT25, BAT26, BAT40, D2S123).E) IGV Trace showing somatic variant NM_000179.2(MSH6) c.2859del p.E953fs
