# Supplementary material for: Clinical Testing for Mismatch Repair in Neoplasms Using Multiple Laboratory Methods
Source: Cancers (Basel). 2022 Sep 20;14(19):4550. doi: 10.3390/cancers14194550 (PMC9559284; doi:10.3390/cancers14194550)
Supplement: Supplementary file 1 [file cancers-14-04550-s001.zip › cancers-1836268-supplementary/SupplementaryDataYanget2022/Supplementary ResultsRKYGATv2.0 - Copy.pdf]

SUPPLEMENTARY RESULTS WITH CASE LEVEL ANNOTATION (SEE ALSO SUPPLEMENTARY TABLE 1)

Mismatch repair deficiency with PCR and IHC in solid neoplasms

Among 706 neoplasms, three major group of neoplasms [colorectal carcinoma (n=316), endometrial carcinoma (n=133), urothelial carcinoma (n=103)] constitute 78% of neoplasms in the study (Table 1). Fifty-eight (8.2%) of 706 neoplasms have MSI-High by PCR and/or dMMR by IHC. With regards to IHC, 49 neoplasms had dMMR (6.9%) and with regards to PCR, 53 had neoplasms have MSI (7.5%)

Typical MMR loss patterns, such as loss of MLH1/PMS2 (n=29) and loss of MSH2/MSH6 (n=13) made up of 79% of cases (42 out of 53) with dMMR loss (Table 2), but atypical dMMR loss patterns such as loss of MLH1/MSH2 (Case #448), isolated losses of MSH2 (Cases #8, #433, #480), MSH6 (Cases #217, #234, #248, #271) and PMS2 (#47, #56, #567) were also observed (Supplementary Table 1).

IHC and MSI analysis are concordant in most neoplasms

Of the 706 analyzed neoplasms, 688 neoplasms (98%) had concordant results: MSI-H/dMMR (n=44), MSS/pMMR (n=625), MSI-L/pMMR(n= 19). Of remaining 18 neoplasms, nine (1.27%) had a major and nine (1.27%) had a minor discordance (Table 2).

Forty-four neoplasms with MSI-H/dMMR comprised 90% of the MSI-H neoplasms (n=49). Five cases out of 49 cases had discordant results. Three cases (#507, #633, #658) had MSI-H/Intact MMR protein expression, which is regarded as a Major Discordance and two cases (#36, #265) had MSI-H/Equivocal MMR protein expression, which is regarded as Minor Discordance (Table 2, Table 3, Supplementary Table 1).

Forty-four neoplasms with MSI-H/dMMR comprised 83% of the dMMR neoplasms (n=53) (Table 2). Nine cases (17%) out of 53 cases had discordant results. Six of 9 discordant cases had major discordance: Three patients (#365, #534, #621) had MSS/Loss of MSH2/MSH6 expression; two patients #217, #271) with MSS/Loss of MSH6 expression and one patient (#554) had MSS/Loss of MLH1/PMS2 expression. Three of 9 discordant cases had a Minor Discordance: Two of these patients (#129, #260) had MSI-L/Loss of MLH1 and PMS2. For these two cases, MLH1 promoter methylation was also observed. One patient (#248) had MSI-L/Loss of MSH6 Discordance (Tables 1-3, Supplementary Table 1).

Concordance rate with MSI for neoplasms with loss of MLH1/PMS2 and loss of MSH6/MSH2 was 90% (26 of 29 cases) and 77% (3 of 13 cases), respectively. For isolated losses of MSH2 and PMS2, this rate was 100%. In contrast, only 25% of neoplasms with isolated MSH6 loss (n=4) had MSI-H by PCR (Table 2).

When stratified by tumor histology, the discordance rate shows some mild variation with colorectal tumors and urothelial tumors being the lowest and neuroendocrine and prostate neoplasms are the highest (Table 1).

Null mutations of MMR genes (and *MLH1* gene promoter methylation) is attributable to most incidents of dMMR

Thirty of 53 neoplasms (57%) with dMMR had a somatic null mutation of MMR gene or a methylation of promoter of *MLH1* gene, whereas only 7 of 653 (1.1%) with normal/equivocal dMMR IHC patterns had a somatic null MMR gene mutation or *MLH1* methylation ($p<0.001$) (Table 4, Supplementary Table 1).

Out of 29 neoplasms with loss of MLH1 and PMS2 expression, 20 neoplasms (69%) had an attributable epigenetic *MLH1* methylation (n=19) and/or somatic null *MLH1* mutation (n=2) (Table 5, top panel). Nineteen neoplasms had a methylation of *MLH1* gene (#6, #16, #17, #62, #80, #107, #108, #116, #129, #148, #233, #256, #260, #268, #270, #310, #382, #553, #706) (Supplementary Table 1). Two of the neoplasms (#6, #16) with *MLH1* methylation had also a somatic pathogenic *MSH6* null or *MLH1* null mutation, respectively. One of the 19 neoplasms with *MLH1* methylation (#17) had a somatic *PMS2* VUS mutation. Among 10 remaining cases with loss of MLH and PMS2 expression, but no documented *MLH1* methylation, one case (#559) had a somatic pathogenic *MLH1* null mutation and one patient (#526) had a germline pathogenic *MSH6* null gene mutation. The other eight cases had no documented germline or somatic mutations. Only two of the 8 cases (#515 and #517) were straight negative for *MLH1*

methylation and MMR gene mutation studies. The remaining 6 of 8 cases had very limited studies for *MLH1* gene. Four cases (#405, #454, #469, #554) had no *MLH1* methylation gene studies and other two of six cases had (#201, #228) very limited somatic MMR gene studies (Table 4, Supplementary Table 1).

Of the 13 neoplasms with loss of MSH2 and MSH6 expression, six (46%) had an attributable somatic pathogenic *MSH2* null (n=5) or *MSH6* null mutation (n=1; #677 Table 4). One of 5 five cases with somatic *MSH2* null mutations (#525) had one pathogenic somatic null *MSH2* and one null *MSH2* germline pathogenic variant (Table 5, bottom panel, Supplementary Table 1). Two of 5 somatic *MSH2* null cases (#49, #72) had a single somatic *MSH2* null pathogenic mutation, and the other two of 5 somatic *MSH2* null cases had more than one somatic *MSH2* mutations: One case (#15) had two *MSH2* null and one *MSH2* VUS; and one case (#53) had two somatic *MSH2* (one null, one VUS). One case (#578) had a likely pathogenic somatic *MSH2* mutation. Remaining six of 13 cases (#135, #355, #365, #399, #534, #621) had no somatic mutations in *MSH2* or *MSH6* genes (full gene study performed for all these cases). Of note, one of the six remaining cases (#365) had a germline *MSH6* VUS.

For 11 neoplasms with atypical IHC patterns (isolated loss of MMR proteins or combined MLH1/MSH2 loss), two of three cases (67%) with an isolated PMS2 loss had an underlying *PMS2* null mutations: Cases #47 had both a somatic and germline *PMS2* null mutation (and a VUS *MSH6*), case #56 had a somatic *PMS2* null mutation only. Regarding the third case with isolated PMS2 loss, case #567 had only a somatic *PMS2 VUS* mutation. Two of 3 cases (66%)

with isolated loss of MSH2 expression had an underlying germline *MSH2* null mutations: Case #8 had a germline *MSH2* null mutation (multiple exon deletions) and two somatic *MSH2* mutations (one null and one VUS), and case #480 had a germline *MSH2* null mutation. Case #433 had no *MSH2* mutation but had a somatic *MSH6* VUS mutation. Among 4 cases with isolated loss of MSH6 expression, only one case (25%) (#271) had an attributable somatic null *MSH6* mutation. The other three cases (#217, #234, #248) did not have a documented *MSH6* mutation due to lack of MSH6 mutation analysis. One case (#448) with a unique loss of MLH1 and MSH2 expression had a somatic VUS on the *MSH2* gene (Supplementary Table 1).

Among 653 patients without overt dMMR, loss 598 patients (92%) had no somatic or germline null mutations, variants of potential clinical significance, or *MLH1* promoter methylation (Supplementary Table 1). Among the remaining 55 patients with germline or somatic mutations or MLH1 promoter methylation, 46 of 55 patients (84%) had an only germline (n=30) or somatic VUS (n=16) mutations. Eight patients had a germline *MLH1* VUS (#289, #393, #438, #493, #565, #595, #671, #674); eight patients had a germline *MSH2* VUS (#18, #35, #87, #103, #109, #128, #289, #298, #429); nine patients had a germline *MSH6* VUS (#44, #121, #354, #371 #423, #439, #472, #506, #507); and five patients had a germline *PMS2* VUS (#123, #286, #353, #415, #629). Sixteen patients had a somatic VUS. Three patients had somatic *MLH1* VUS (#232, #577, #625) and seven patients had *MSH2* VUS (#41, #57, #265, #490, #512, #647, #663). Patients #265 and #663 each had two somatic *MSH2* mutations and patient #265 had equivocal results for MSH6 loss as well as MSI-H by PCR. Six patients had somatic *MSH6* VUS (#58, #343, #542, #571, #588, #700) mutations. Patient #542 had three somatic *MSH6* mutations.

The remaining nine cases had at least one pathogenic/likely pathogenic mutation or *MLH1* methylation. Two patients had a likely somatic pathogenic *MSH6* missense mutations (#79, #394). One patient (#633) had an *MLH1* promoter methylation and had MSI-H by PCR. Two patients (#36, #658) had both a germline *MSH6* pathogenic null mutation and somatic null *MSH6* mutation. Both cases were MSI-H by PCR and case #36 had equivocal IHC results for MSH2/MSH6. Four patients each had a single pathogenic somatic null mutation: *MSH2* null (cases #239, #562), *MSH6* null (#522) and *PMS2* null (#570).

<u>Null mutations of MMR genes and MLH1 gene promoter methylation is attributable to most incidents of MSI-High</u>

<u>MSI High</u>

Thirty of 49 neoplasms (61%) with MSI-H status by PCR had a somatic null mutation of MMR gene or a methylation of promoter of *MLH1* gene, whereas only 7 of 657 (1.1%) with MSS/MSI-L patterns had a null MMR gene mutation or *MLH1* methylation ($p<0.001$) (Table 4, Supplementary Table 1) Among the 49 cases with MSI-H, 18 cases (37%) (#6, #16, #17, #62, #80, #107, #108, #116, #148, #233, #256, #268, #270, #310, #382, #553, #633, #706) had *MLH1* promoter methylation. In addition to *MLH1* promoter methylation, cases #6, #16, #17 ha somatic pathogenic null *MSH6*, null *MLH1* and VUS *PMS2* mutations, respectively (Supplementary Table 1).

Somatic null mutations of MMR gene were observed in 14 (29%) of 49 cases. Two cases with *MLH1* null mutation had a *MLH1* promoter methylation (#16) and a second *MLH1* VUS (#559) mutation, respectively. Six cases had a *MSH2* null mutation. Among these six, only cases #49 and #72 had a single isolated somatic *MSH2* null mutation. The other four cases had multiple*MSH2* somatic and/or germline mutations. In addition to the somatic *MSH2* null mutations, Cases #8 and #525 cases had also a germline null pathogenic *MSH2* mutations. Cases #15 and #53 had a second pathogenic *MSH2* mutation. Four cases had a somatic pathogenic MSH6 null mutation. None of these four cases demonstrated isolated *MSH6* somatic events. One case (#6) had a *MLH1* promoter methylation. Two cases (#36, #658) had germline null pathogenic mutation. One case (#677) had additional somatic *MSH6* mutation. Among two cases with somatic pathogenic *PMS2* null mutations, one case (#47) had an additional germline pathogenic null *PMS2* mutation, and one case (#57) had an isolated somatic *PMS2* null mutation.

Six of the 49 MSI-H cases (12%) had somatic missense MMR gene mutations only. One case (#578) had a likely pathogenic somatic *MSH2* mutation. The remining five cases had somatic VUS mutations. Two of the five cases (#265, #448) had *MSH2* somatic VUS mutations, one case had a *MSH6* somatic VUS mutation (#433), and two cases (#17, #567) had *PMS2* somatic VUS respectively. Case #265 had two *MSH2* somatic VUS mutations and case #17 had *MLH1* promoter methylation in addition to somatic *PMS2* VUS mutation. Three of the 49 cases had germline variants only. Cases #480 and #526 had a germline pathogenic *MSH2* null and *MSH6* null mutations, respectively, only. In contrast, case #507 had a *MSH6* germline VUS mutation. Among 11 cases without documented MLH1 methylation and NGS mutations and of the 49

cases with MSI-H, only two neoplasms (#515 and #517) are negative for *MLH1* methylation and MMR gene mutation studies. Seven of the 11 (#135, #234, #355, #399 #405, #454, #469,) neoplasms had no *MLH1* methylation gene studies, and other two of 11 had (#201, #228) had very limited genetic studies. Eleven of 49 cases (22%) had no documented mutations or *MLH1* promoter methylation. Only two of the 11 neoplasms (#515 and #517) were straight negative for *MLH1* methylation and MMR gene mutation studies. The remaining 9 of 11 patients had very limited studies for *MLH1* gene. Seven neoplasms (#135, #234, #355, #399 #405, #454, #469) had no *MLH1* methylation gene studies and other two of nine have (#221, 228) have very limited somatic MMR gene studies.

MSS-L

Among 23 cases with MSI-Low phenotype by PCR, two cases (#129, #260) had *MLH1* methylation; two cases (#57, #512) had somatic *MSH2* VUS mutations, and one case had a germline *MLH1* VUS (#289) mutation (Table 4, Supplementary Table 1). Two patients with MSI-L by PCR (#129, #260) also had *MLH1* promoter methylation.

MSS

Among 634 patients with MSS, 585 patients (92%) had no somatic or germline null mutations or variants of potential clinical significance. Among the remaining 49 patients with mutations, 29 of 49 patients (59%) had an only germline VUS mutations (Table 6, bottom right panel). Seven of 29 patients (#393, #438, #493, #565, #595, #671, 674) had germline *MLH1* VUS mutations; Eight of 29 patients (#18, #35, #87, #103, #109, #128, #298, #429) has germline *MSH2* VUS

mutations; Nine of 29 patients (#44, #121, #354, #365, #371 #423, #439, #472, #506) had germline *MSH6* VUS mutations; and five of 29 patients (#123, #286, #353, #415, #629) had germline *PMS2* VUS mutations (Supplementary Table 1).

Five patients had somatic pathogenic null mutations: *MSH2* null (cases #239, #562), *MSH6* null (#271, #522), and *PMS2* null (#570). Patient #271 with somatic null *MSH6* mutation had also loss of MSH6 protein expression by IHC. Two patients had somatic Likely Pathogenic *MSH2* missense mutations (#79, #394). Thirteen MSS or MSI-L patients had isolated somatic VUS mutations. Three of 13 patients had *MLH1* VUS (#232, #577, #625) and five of 13 patients had *MSH2* VUS (#41, #57, #490, #647, #663). Patients #663 had two somatic *MSH2* VUS mutations. Six of 15 somatic VUS mutation only MSS/MSI-L patients had *MSH6* VUS (#58, #343, #542, #571, #588, #700). Patient #542 had three somatic *MSH6* VUS mutations.

<u>Most of the neoplasms with major discordance do not have documented underlying genetic/epigenetic pathogenic abnormality</u>

Among 9 cases with major discordance between MSI and IHC, only 3 cases (33%) had an underlying pathogenic genetic/epigenetic MMR gene abnormality (Table 4), whereas 37 of 49 cases (76%) with MSI-H and/or dMMR and without major discordance, had an underlying genetic abnormality (p=0.019) (Supplementary Table 1).

Three cases with major discordance and underlying significant genetic mutations were: One patient (#271 with MSS/Loss of MSH6 had a somatic pathogenic *MSH6* mutation; one patient (#633) with MSI-H/Intact MMR expression had a *MLH1* promoter methylation; one patient (#658) with MSI-H/Intact MMR expression had a germline pathogenic *MSH6* null mutation and somatic MSH6 mutation. Twelve patients with dMMR and MSI (High or Low) (#135, #201, #228, #234, #248, #355, #399, #405, #454, #469, #515, #517) did not have a documented underlying somatic genetic/epigenetic *MLH1* methylation aberration or a germline pathogenic abnormality.
